# Association between fluctuations in serum chloride levels and 30-day mortality among critically ill patients: a retrospective analysis

**DOI:** 10.1186/s12871-019-0753-3

**Published:** 2019-05-17

**Authors:** Hyo Jin Kim, Tak Kyu Oh, In-Ae Song, Jae Ho Lee

**Affiliations:** 1 Department of Anesthesiology and Pain Medicine, Inje University Seoul Paik hospital, Seoul, South Korea; 2 Department of Anesthesiology and Pain Medicine, Seoul National University Bundang Hospital, Gumi-ro 173 Beon-gil, Bundang-gu, Seongnam 13620 Korea; 3 Division of Pulmonary and Critical Care Medicine, Department of Internal Medicine, Seoul National University Bundang Hospital, Seongnam, South Korea

**Keywords:** Intensive care unit, Mortality, Sepsis, Critical care, Hyperchloremia

## Abstract

**Background:**

This study investigated the associations of fluctuations in serum chloride (Cl^−^) levels with 30-day mortality after intensive care unit (ICU) admission among critically ill patients.

**Methods:**

We retrospectively analyzed the medical records of adult patients (≥18 years old) admitted to the ICU between January 2012 and December 2017. Positive and negative fluctuations in Cl^−^ were defined as the differences between the Cl^−^ upon ICU admission (baseline Cl^−^) and the maximum and minimum Cl^−^ levels, respectively, measured within 72 h after ICU admission.

**Results:**

The final analysis included 18,825 adult patients. In multivariable Cox regression analyses, the risk of 30-day mortality increased by 8% per 1-mmol L^− 1^ positive fluctuation in Cl^−^ within 72 h (hazard ratio = 1.08, 95% confidence interval: 1.04–1.11, *P* < 0.001). In subgroup analyses, a positive fluctuation in Cl^−^ was associated with increased 30-day mortality among patients with a severe positive cumulative fluid balance (FB, > 10%), normochloremia (97–110 mmol L^− 1^) or hyperchloremia (> 110 mmol L^− 1^) upon ICU admission. Furthermore, a negative fluctuation in the Cl^−^ level during the first 72 h of an ICU stay was associated with a negative cumulative FB (< 0%) or hypochloremia (< 97 mmol L^− 1^) upon ICU admission.

**Conclusions:**

A fluctuation in the Cl^−^ level during the first 72 h of an ICU stay was found to associate independently with increased 30-day mortality among critically ill adult patients. However, the nature of this association differed according to the cumulative FB status or dyschloremia status upon ICU admission.

**Electronic supplementary material:**

The online version of this article (10.1186/s12871-019-0753-3) contains supplementary material, which is available to authorized users.

## Background

Serum chloride (Cl^−^), the most abundant anion in plasma and interstitial fluid, is an important determinant of plasma tonicity [1, 2]. Therefore, the maintenance of Cl^−^ homeostasis is essential to the maintenance of electrolyte and acid-base balances in the body and the regulation of body fluids [1, 3].

Dyschloremia, a common complication observed in critically ill patients with varied etiologies, can be categorized as hypochloremia (i.e., a serum Cl^−^ level below the normal range) or hyperchloremia (a serum Cl^−^ level above the normal range) [4]. Several studies of the role of Cl^−^ in critically ill patients have evaluated the association between dyschloremia and mortality in this population [4]. For example, both hyperchloremia [3, 5–7] and hypochloremia have been reported to associate with increased hospital mortality among critically ill patients [8–10]. These findings suggest that in this patient population, the association of serum Cl^−^ levels with mortality among critically ill patients yields a U-shaped curve in which both hypo- and hyperchloremia are detrimental to patient survival [11]. Still, this issue remains controversial.

As the Cl^−^ level is strongly affected by fluid resuscitation, previous studies of the association between dyschloremia and mortality in critically ill patients have focused on increases in Cl^−^ loading and levels [12–15]. In critically ill patients, however, the Cl^−^ level may decrease after intensive care unit (ICU) admission due to a loss of active Cl^−^ from the gastrointestinal tract, impaired renal Cl^−^ reabsorption, and an infusion of hypotonic fluid [1, 16]. Accordingly, overall fluctuations in Cl^−^ levels, including positive or negative fluctuations, must be considered when determining the association of Cl^−^ levels with mortality in critically ill patients.

Therefore, this study aimed to examine the association between both positive and negative fluctuations in Cl^−^ levels and 30-day mortality among critically ill patients admitted to the ICU. As the impact of fluctuations in Cl^−^ levels after ICU admission could be affected by the cumulative fluid balance (FB) or dyschloremia upon ICU admission, we also investigated whether these factors might have different effects on the association between fluctuations in Cl^−^ levels and 30-day mortality.

## Methods

### Design and ethical statement

This retrospective observational study was approved by the Institutional Review Board (IRB) of Seoul National University Bundang Hospital (IRB approval number: B-1806/474–105; approval date: 2018. 6. 11), which waived the requirement to obtain informed consent from the subjects. All data were collected by medical record technicians from the medical informatics team of the institution who were blinded to the purpose of the study.

### Patients

The medical records of all adult patients aged ≥18 years who were admitted to the ICU between January 2012 and December 2017 were reviewed. If a patient was admitted to the ICU two or more times during the study period, only the last admission (which may have been the most severe) was included in the analysis. Patients with incomplete or missing medical records related to Cl^−^ levels were excluded from the analysis.

### Fluctuations in cl^−^ during the 72-h period after ICU admission (main independent variable)

In this study, the Cl^−^ level upon ICU admission (baseline Cl^−^) was defined as the first Cl^−^ level measured within 24 h after ICU admission. Positive and negative fluctuations in Cl^−^ were defined as the differences between the baseline Cl^−^ and the maximum and minimum Cl^−^ levels, respectively, measured within 72 h after ICU admission. If a patient died within 72 h after ICU admission, the fluctuation in Cl^−^ was calculated using data recorded in the ICU prior to death.

### Definitions of normo-, hypo-, and hyperchloremia on ICU admission

We used the baseline Cl^−^ measured as defined above to diagnose dyschloremia at the time of ICU admission. Cl^−^ statuses were defined as follows: normochloremia, 97–110 mmol L^− 1^; hypochloremia, < 97 mmol L^− 1^; and hyperchloremia, > 110 mmol L^− 1^.

### Cumulative fluid balance (%) during the 72-h period after ICU admission

The weight-based cumulative FB (%) was calculated using the following formula suggested in previous studies [17, 18]: (Total fluid input – output in L) ×  100% × (hospital admission weight in kg)^− 1^. Fluid input was defined as all types of intravenous and enteral fluids used for maintenance or resuscitation. Fluid output was defined as all types of eliminated and removed fluids (e.g., drainage, rectal, orogastric, nasogastric, and urine output). If a patient died within 72 h after ICU admission, the cumulative FB was calculated from ICU admission until death. All patients were subsequently categorized based on their cumulative FB into the following categories: positive FB (≥5%), even FB (0–5%), or negative FB (< 0%). In addition, the positive FB group was divided into 2 groups: mild or moderate positive FB (5–10%) and severe FB (> 10%), based on previous definitions [19].

### Other measurements (potential covariates)

The patients’ physical characteristics [sex, age (years), and body mass index (kg m^− 2^)]; Acute Physiology and Chronic Health Evaluation II scores; comorbidities upon ICU admission [hypertension, diabetes mellitus, ischemic heart disease, cerebrovascular disease, chronic obstructive lung disease, liver disease (liver cirrhosis, hepatitis, fatty liver), dyslipidemia, chronic kidney disease, anemia, cancer]; hospital admission through the emergency department; and admitting department (internal medicine, neurologic center, post-cardiothoracic surgery, or post-other surgery) were obtained from the database. Packed red blood cell transfusion, renal replacement therapy, vasopressor infusion, and fluid administration (NaCl 0.9%, balanced crystalloid, and hydroxyethyl starch in ml) within 72 h after ICU admission were recorded as reflective of treatment. Additionally, the number of Cl^−^ measurements within 72 h after ICU admission was also collected.

### 30-day mortality (dependent variable)

In this study, 30-day mortality was defined as death within 30 days of the ICU admission date. We obtained approval from the Ministry of the Interior and Safety in South Korea to determine the exact date of death of each patient, including those who were discharged from the hospital. We were able to obtain the exact dates of death for all patients as of May 16, 2018.

### Aim

This study assessed associations of fluctuations (positive or negative) in the Cl^−^ levels within 72 h after ICU admission with 30-day mortality after ICU admission. Additionally, we investigated whether this association might differ according to the cumulative FB status or dyschloremia status upon ICU admission.

### Statistical analysis

Initially, the relationship between a positive or negative fluctuation in Cl^−^ levels within 72 h after ICU admission (continuous variable) and 30-day mortality was first examined using a restricted cubic spline (Additional file 3: Figure S1 and Additional file 4: Figure S2, respectively). Next, we subdivided both positive and negative fluctuations in Cl^−^ into two groups each, using a cut-off point of 10 mmol L^− 1^. First, as the end points of the U-shaped curve of 30-day mortality associated with both positive and negative fluctuations in Cl^−^ occurred at approximately 10 mmol L^− 1^ in the cubic spline analyses, this stratification could maximize the impact of fluctuations in Cl^−^ on 30-day mortality. Second, a previous similar study suggested that 30-day mortality was lowest among critically ill patients with systemic inflammatory response syndrome who exhibited a 0–10 mmol L^− 1^ increase in Cl^−^ [5].

We next examined the independent associations of each of the covariates with 30-day mortality using a univariable Cox regression analysis (Additional file 1: Table S1). Covariates that received a *P* < 0.1 in the univariable analysis were included in the final multivariable Cox regression analysis for covariate adjustment. The main independent variable, fluctuation in Cl^−^, was included as both a continuous and categorical variable (> 10 mmol L^− 1^ or ≤ 10 mmol L^− 1^) in different models to avoid multicollinearity between variables. Then, we investigated the interactions of cumulative FB groups or dyschloremia upon ICU admission with the effects of fluctuations in Cl^−^ on 30-day mortality. After confirming these interactions, we performed a subgroup analysis according to the previously described cumulative FB groups and dyschloremia upon ICU admission using the Bonferroni correction to reduce type 1 errors in multiple comparisons [20]. The results of the Cox regression analysis are presented as hazard ratios (HRs) with 95% confidence intervals (CIs), and a log-minus-log plot was used to confirm that the central assumptions of the Cox proportional hazard model were satisfied in each multivariable model.

All data were analyzed using IBM SPSS version 24.0 (IBM, Armonk, NY, USA). A *P* < 0.05 was considered statistically significant. In the subgroup analyses, a *P* < 0.012 or < 0.017 was considered statistically significant in the analyses conducted according to FB groups or dyschloremia upon ICU admission, respectively, after applying the Bonferroni correction.

## Results

**Fig. 1:**
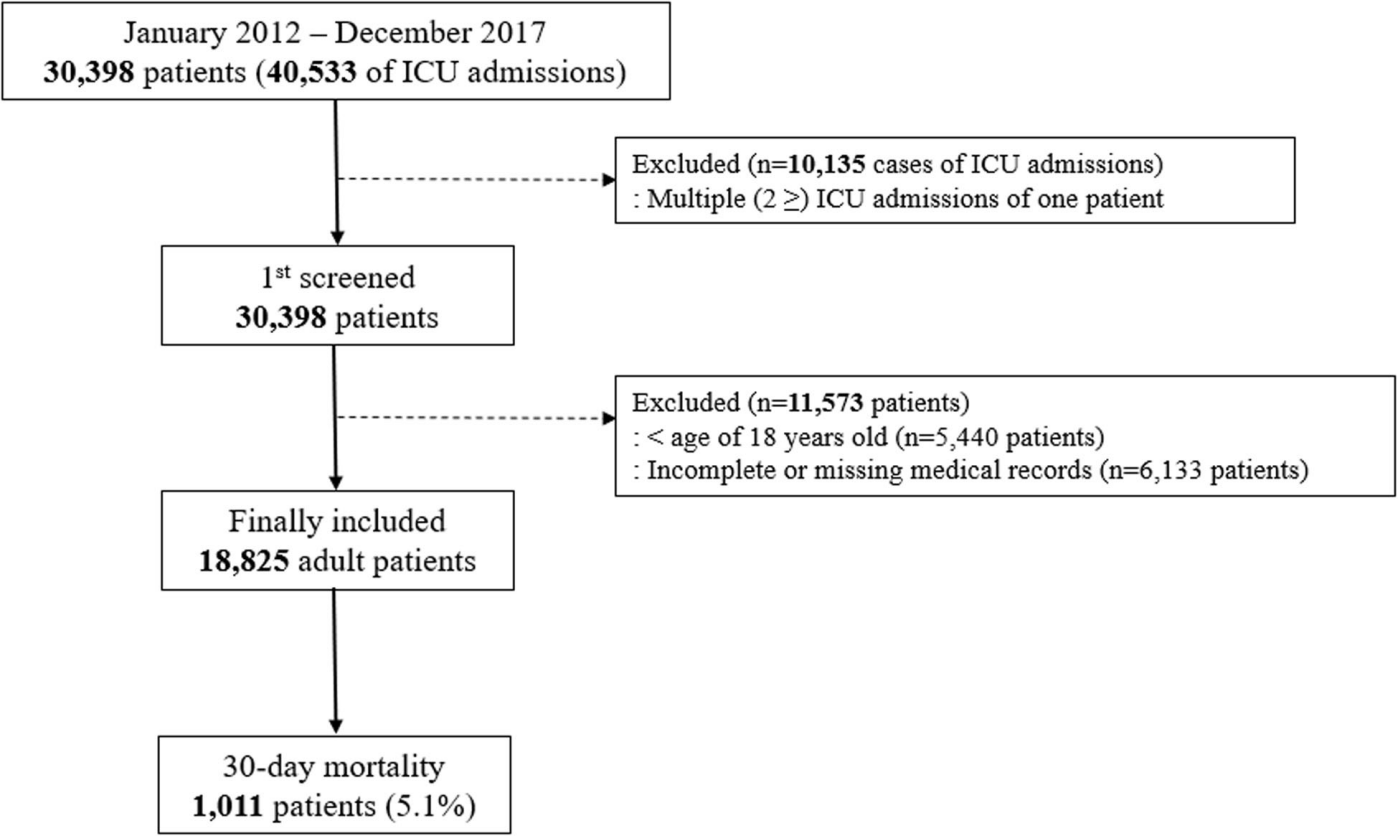
Flow chart of patient selection. ICU, intensive care unit

A total of 40,533 ICU admissions were recorded between January 2012 and December 2017. Of these, 10,135 cases involving patients with more than two admissions were excluded. Among the 30,398 admissions consequently included in the initial screening, 5440 involving patients younger than 18 years and 6133 with incomplete or missing medical records relevant to this analysis were excluded. The final analysis sample included 18,825 adult patients, among whom 1011 (5.1%) died within 30 days (Fig. 1). The baseline characteristics of all patients included in the analysis are shown in Table 1.

**Table 1:** Baseline characteristics of adults patients admitted to the ICU during 2012–2017

Variable	Total (18,825)	Mean	SD
Sex: male	10,839 (57.6%)		
Age, years		63.7	16.0
Body mass index, kg m^−2^		23.6	3.9
APACHE II		20.3	9.8
Comorbidities at ICU admission			
Hypertension	8160 (43.3%)		
Diabetes mellitus	1673 (8.9%)		
Ischemic heart disease	447 (2.4%)		
Cerebrovascular disease	856 (4.5%)		
Chronic obstructive lung disease	836 (4.4%)		
Liver disease (LC, hepatitis, fatty liver)	603 (3.2%)		
Dyslipidemia	1191 (6.3%)		
Chronic kidney disease	3236 (17.2%)		
Anemia (Hb < 10 g dl^−1^)	6919 (36.8%)		
Cancer	4001 (21.3%)		
Characteristics of ICU admission			
Admission through emergency department	10,950 (58.2%)		
Admitting department			
Internal medicine	3851 (20.5%)		
Neurologic center	4770 (25.3%)		
Post-cardiothoracic surgery	5765 (30.6%)		
Post-other surgery	4438 (23.6%)		
Length of ICU stay, day		3.4	10.6
Length of hospital stay, day		13.8	20.8
pRBC transfusion within 72 h after ICU adm	8294 (44.1%)		
RRT within 72 h after ICU adm	458 (2.4%)		
Vasopressor infusion within 72 h after ICU adm	3433 (18.2%)		
^a^Cumulative fluid balance within 72 h after ICU adm		3.2%	4.7%
Negative (< 0%)	3933 (20.9%)		
Even (0–5%)	9680 (51.4%)		
Positive, Mild to moderate (5–10%)	3889 (20.7%)		
Positive, Severe (> 10%)	1323 (7.0%)		
Fluid administration for 72 h after ICU admission			
NaCl 0.9%, ml		1729.1	2097.8
Balanced crystalloid, ml		521.9	871.2
Hydroxyethyl starch, ml		78.3	249.8
Serum chloride (Cl^−^) in ICU, mmol L^− 1^			
The number of measurements for 72 h after ICU adm		3.3	0.9
Cl^−^ on ICU admission (baseline Cl^−^)		103.7	5.6
Normochloremia on ICU adm (97–110 mmol L^−1^)	11,861 (63.0%)		
Hypochloremia on ICU adm (< 97 mmol L^− 1^)	1753 (9.3%)		
Hyperchloremia on ICU adm (> 110 mmol L^− 1^)	5211 (27.7%)		
Maximum Cl^−^ within 72 h after ICU adm		107.8	5.7
Minimum Cl^−^ within 72 h after ICU adm		100.8	5.1
^b^Positive fluctuation of Cl^−^ for 72 h after ICU adm		4.1	4.1
> 10 mmol L^−1^	1243 (6.6%)		
^c^Negative fluctuation of Cl^−^ for 72 h after ICU adm		2.9	3.5
> 10 mmol L^−1^	682 (3.6%)		
30-day mortality after ICU admission	1011 (5.1%)		

*ICU* intensive care unit, *APACHE* Acute Physiology and Chronic Health Evaluation, *adm* admission, *LC* liver cirrhosis, *Hb* hemoglobin, *pRBC* packed red blood cells, *RRT* renal replacement therapy

^a^Cumulative fluid balance: (Total input fluid – total output fluid, L) × 100 x weight in admission (kg)^−1^

^b^Positive fluctuation in Cl^−^: (Maximum Cl^−^ – Preadmission Cl^−^) for 72 h after ICU admission

^c^Negative fluctuation in Cl^−^: (Preadmission Cl^−^ – Minimum Cl^−^) for 72 h after ICU admission

### Fluctuations in cl^−^ levels within 72 h after ICU admission and 30-day mortality

The results of the univariable and multivariable Cox regression analyses are presented in Additional file 1: Table S1. The multivariable Cox regression analysis (Table 2) revealed that each 1-mmol L^− 1^ positive fluctuation in the Cl^−^ level was associated with an 8% increase in the risk of 30-day mortality (HR = 1.08, 95% CI: 1.04–1.11, *P* < 0.001; model 1). When patients exhibiting positive fluctuations were stratified using a cut-off value of a 10-mmol L^− 1^ positive fluctuation in Cl^−^ levels within 72 h of ICU admission, those with fluctuations above this value (> 10 group) had a 3.37-fold higher risk of 30-day mortality, compared to those with fluctuations below this value (≤10 group) (HR = 3.37, 95% CI: 2.01–5.67, *P* < 0.001; model 2). However, a negative fluctuation in Cl^−^ was not associated significantly with the risk of 30-day mortality (*P* = 0.313 in model 1 and *P* = 0.883 in model 2).

**Table 2:** Multivariable Cox regression analysis of 30-day mortality after ICU admission according to positive and negative fluctuations in Cl^−^ levels within 72 h after ICU admission

Variables	Multivariable model	*P*
	Hazard ratio (95% CI)	
Model 1		
^a^Positive fluctuation in Cl^−^ (1 mmol L^− 1^ increase)	1.08 (1.04, 1.11)	< 0.001
^b^Negative fluctuation in Cl^−^ (1 mmol L^− 1^ increase)	1.03 (0.98, 1.08)	0.313
Cumulative FB group^c^		
Even (0–5%)	1	(< 0.001)
Negative (< 0%)	0.97 (0.72, 1.32)	0.868
Positive, Mild to moderate (5–10%)	2.01 (1.57, 2.57)	< 0.001
Positive, Severe (> 10%)	4.06 (3.10, 5.32)	< 0.001
Dyschloremia at ICU admission		
Normochloremia (97–110 mmol L^− 1^)	1	(< 0.001)
Hypochloremia (< 97 mmol L^− 1^)	1.99 (1.57, 2.51)	< 0.001
Hyperchloremia (> 110 mmol L^− 1^)	0.78 (0.60, 1.00)	0.054
Model 2		
^a^Positive fluctuation of Cl^−^ > 10 mmol L^− 1^	3.37 (2.01, 5.67)	< 0.001
^b^Negative fluctuation of Cl^−^ > 10 mmol L^− 1^	0.94 (0.38, 2.30)	0.883

*ICU* intensive care unit, *FB* fluid balance

^a^Positive fluctuation in Cl^−^: (Maximum Cl^−^ – Preadmission Cl^−^) for 72 h after ICU admission

^b^Negative fluctuation in Cl^−^: (Preadmission Cl^−^ – Minimum Cl^−^) for 72 h after ICU admission

^c^Cumulative fluid balance (%): (Total input fluid – total output fluid, L) × 100 x weight at admission (kg)^− 1^

### Subgroup analysis according to cumulative fluid balance and dyschloremia on ICU admission

As the interactions of FB groups and dyschloremia upon ICU admission with fluctuations in Cl^−^ affected 30-day mortality (Additional file 2: Table S2), we performed subgroup analyses (Table 3). Among patients in the negative FB group (< 0%), a > 10-mmol L^− 1^ negative fluctuation in Cl^−^ was associated with a 3.04-fold increase in 30-day mortality, compared to those with a negative fluctuation of ≤10 mmol L^− 1^ (HR = 3.04, 95% CI: 1.32–7.03, *P* = 0.009). Among patients in the severely positive FB group (> 10%), a > 10 mmol L^− 1^ negative fluctuation in Cl^−^ was associated with a 2.07-fold increase in 30-day mortality, compared to those with a negative fluctuation of ≤10 mmol L^− 1^ (HR = 2.07, 95% CI: 1.35–3.16, *P* = 0.001).

**Table 3:** Subgroup analysis according to the cumulative fluid balance group within 72 h after ICU admission and dyschloremia upon ICU admission

Variables	Multivariable model	
	Hazard Ratio (95% CI)	*P* ^*^
^a^Cumulative FB group (subgroup analysis 1)		
Even (0–5%): 9680		
^b^Positive fluctuation of Cl^−^ > 10 mmol L^− 1^	1.76 (1.12, 2.78)	0.015
^c^Negative fluctuation of Cl^−^ > 10 mmol L^− 1^	1.01 (0.46, 2.21)	0.986
Negative (< 0%): 3933		
^b^Positive fluctuation of Cl^−^ > 10 mmol L^− 1^	1.34 (0.64, 2.83)	0.441
^c^Negative fluctuation of Cl^−^ > 10 mmol L^− 1^	**3.04** (1.32, 7.03)	**0.009**
Positive, Mild to moderate (5–10%): 3889		
^b^Positive fluctuation of Cl^−^ > 10 mmol L^− 1^	1.31 (0.79, 2.17)	0.289
^c^Negative fluctuation of Cl^−^ > 10 mmol L^− 1^	1.02 (0.50, 2.05)	0.967
Positive, Severe (> 10%): 1323		
^b^Positive fluctuation of Cl^−^ > 10 mmol L^− 1^	**2.07** (1.35, 3.16)	**0.001**
^c^Negative fluctuation of Cl^−^ > 10 mmol L^− 1^	1.30 (0.80, 2.11)	0.287
Dyschloremia on ICU admission (subgroup analysis 2)		*P* ^**^
Normochloremia (97–110 mmol L^− 1^): 11,861		
^b^Positive fluctuation of Cl^−^ > 10 mmol L^− 1^	**2.59** (1.74, 3.86)	**< 0.001**
^c^Negative fluctuation of Cl^−^ > 10 mmol L^− 1^	1.00 (0.61, 1.66)	0.993
Hypochloremia (< 97 mmol L^− 1^): 1753		
^b^Positive fluctuation of Cl^−^ > 10 mmol L^− 1^	1.03 (0.69, 1.53)	0.903
^c^Negative fluctuation of Cl^−^ > 10 mmol L^− 1^	**4.66** (1.39, 15.65)	**0.012**
Hyperchloremia (> 110 mmol L^− 1^): 5211		
^b^Positive fluctuation of Cl^−^ > 10 mmol L^− 1^	**1.75** (1.11, 2.74)	**0.014**
^c^Negative fluctuation of Cl^−^ > 10 mmol L^− 1^	1.50 (0.93, 2.40)	0.095

A P^*^ < 0.012 and P^**^ < 0.017 indicate statistical significance after Bonferroni correction in analyses of four subgroups

*ICU* intensive care unit, *FB* fluid balance

^a^Cumulative fluid balance (%): (Total input fluid – total output fluid, L) × 100 x weight at admission (kg)^− 1^

^b^Positive fluctuation in Cl^−^: (Maximum Cl^−^ – Preadmission Cl^−^) for 72 h after ICU admission.

^c^Negative fluctuation in Cl^−^: (Preadmission Cl^−^ – Minimum Cl^−^) for 72 h after ICU admission.

In addition, a > 10 mmol L^− 1^ positive fluctuation in Cl^−^ was associated with 2.59-fold (HR = 2.59, 95% CI: 1.74–3.86, *P* < 0.001) and 1.75-fold increases (HR = 1.75, 95% CI: 1.11–2.74, *P* = 0.014) in 30-day mortality among patients with normochloremia and hyperchloremia upon ICU admission, respectively. On the other hand, a > 10 mmol L^− 1^ negative fluctuation in Cl^−^ was associated with a 4.66-fold increase in 30-day mortality (HR-4.66, 95% CI: 1.39–15.65, *P* = 0.012) among patients with hypochloremia upon ICU admission.

**Fig. 2:**
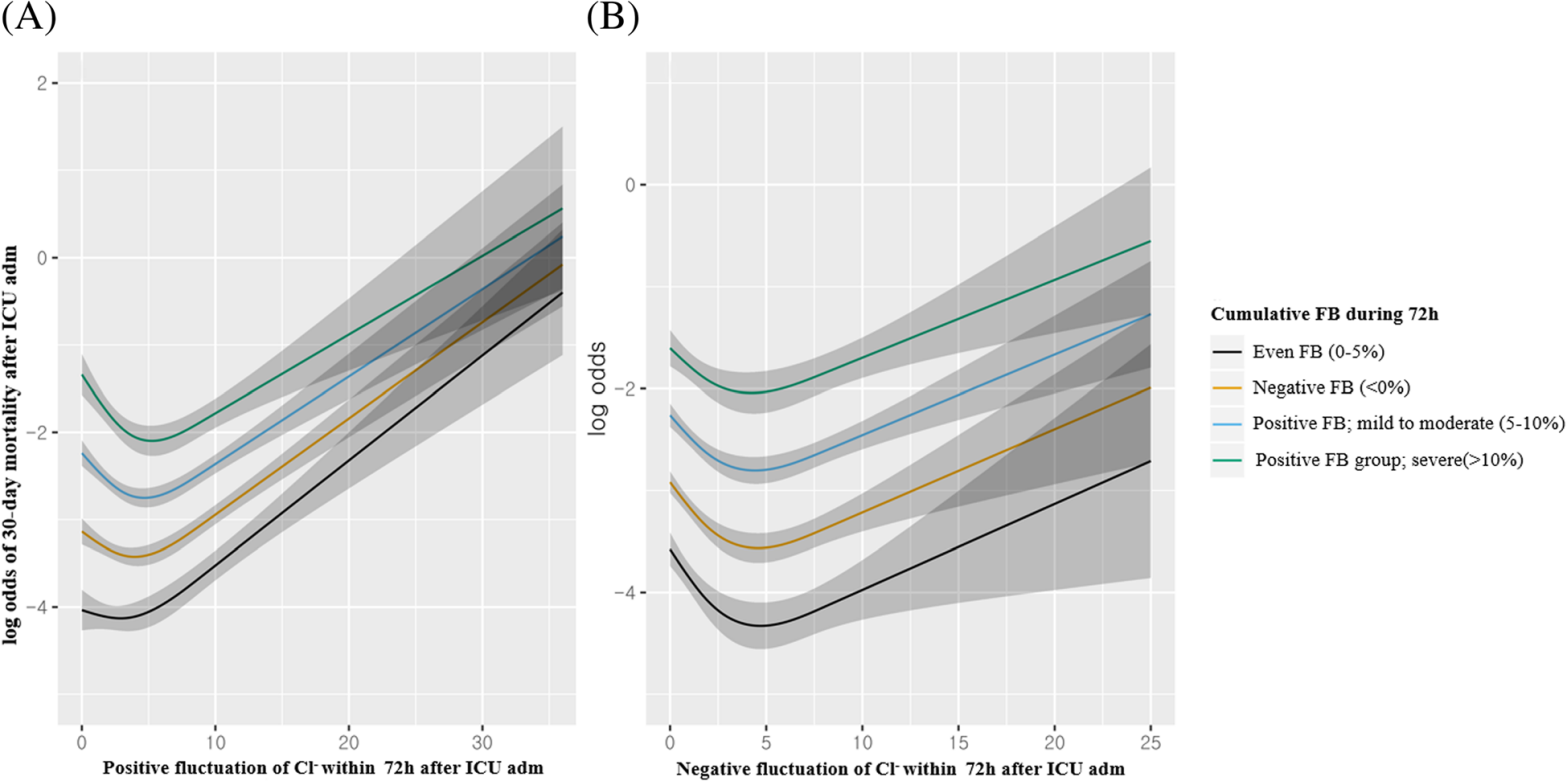
Restricted cubic spline analysis of 30-day mortality with positive (**a**) and negative (**b**) fluctuations in Cl^−^ within 72 h according to the cumulative fluid balance during 72 h after ICU admission. Cumulative fluid balance (%) was calculated as: (Total input fluid – total output fluid, L) × 100 x weight in admission (kg)^− 1^. Positive fluctuation in Cl^−^: Maximum Cl^−^ level within 72 h after ICU admission – Cl^−^ level upon ICU admission. Negative fluctuation in Cl^−^: Cl^−^ level upon ICU admission – minimum Cl^−^ level within 72 h after ICU admission

**Fig. 3:**
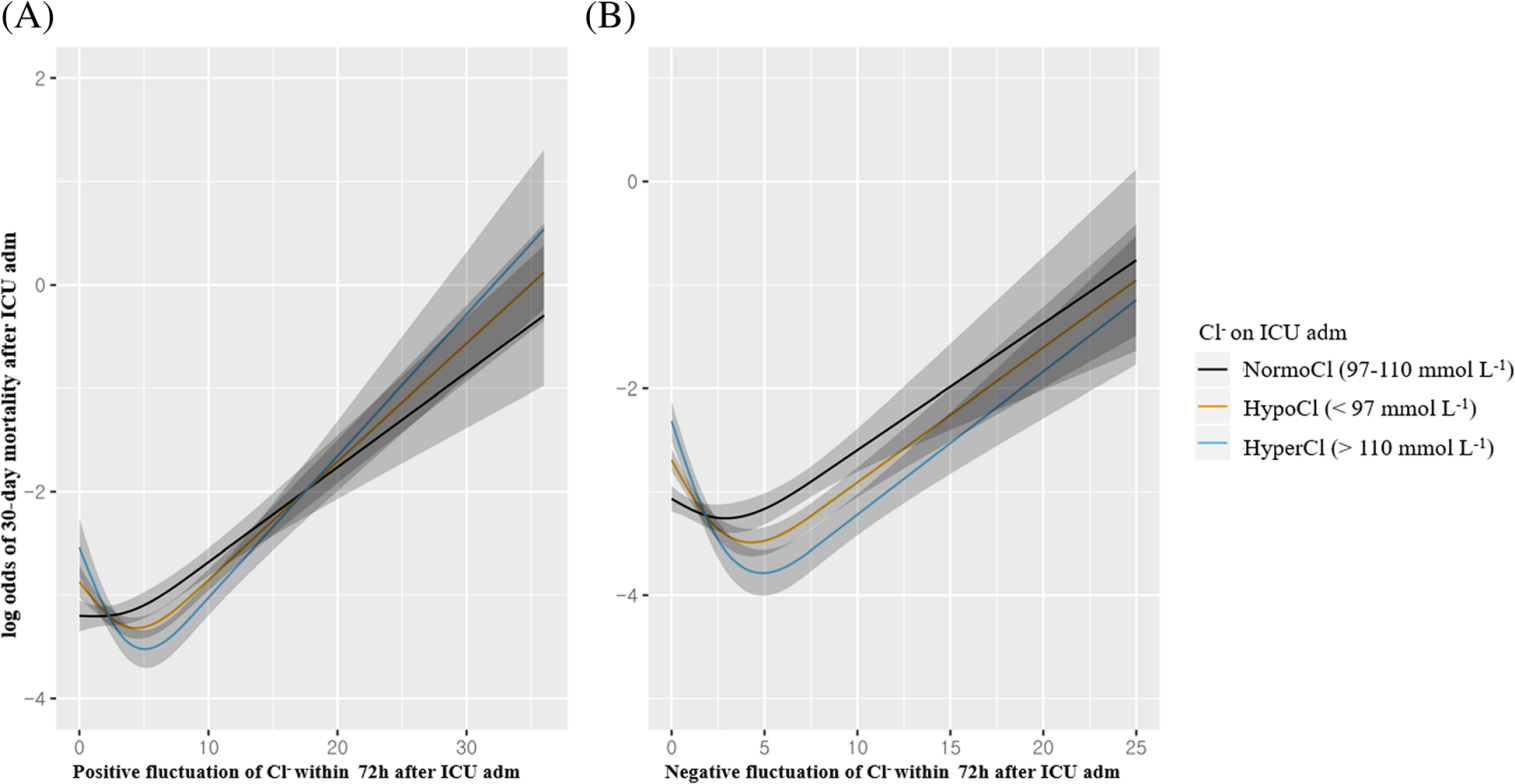
Restricted cubic spline analysis of 30-day mortality with positive (**a**) and negative (**b**) fluctuations in Cl^−^ within 72 h according to dyschloremia upon ICU admission. Normochloremia: 97–110 mmol L^− 1^, Hyperchloremia: > 110 mmol L^− 1^, Hypochloremia: < 97 mmol L^− 1^. Positive fluctuation in Cl^−^: Maximum Cl^−^ level within 72 h after ICU admission – Cl^−^ level upon ICU admission. Negative fluctuation in Cl^−^: Cl^−^ level upon ICU admission – minimum Cl^−^ level within 72 h after ICU admission

Figure 2a and b depict the restricted cubic splines for 30-day mortality with positive and negative fluctuations in Cl^−^, respectively, according to cumulative FB groups. Figure 3a and b depict the restricted cubic splines for 30-day mortality with positive and negative fluctuations in Cl^−^, respectively, according to the dyschloremia status upon ICU admission.

## Discussion

Our findings from a retrospective sample of ICU patients generally suggest that a positive fluctuation in Cl^−^ levels within 72 h after ICU admission is associated with a greater risk of 30-day mortality. By contrast, no similar association was observed with a negative fluctuation in Cl^−^ levels. However, this association might differ according to the cumulative FB status or dyschloremia status upon ICU admission. In these subgroup analyses, a positive fluctuation in Cl^−^ was associated with an increased risk of 30-day mortality among patients in the severe positive FB group (> 10%) and those with normochloremia or hyperchloremia upon ICU admission, whereas a negative fluctuation in Cl^−^ was associated with an increased risk of 30-day mortality among patients with a negative FB (< 0%) or hypochloremia upon ICU admission. These findings are valuable because we evaluated the effects of both positive and negative fluctuations in Cl^−^ on post-ICU admission mortality after considering the interactions of the cumulative FB and dyschloremia status upon ICU admission. Our results further suggest that in the ICU, more severely ill patients are at an increased risk of dysregulated Cl^−^ handling because they are highly likely to receive more interventions and larger amounts of crystalloid and to experience altered fluid handling.

First, the observation that a positive fluctuation in Cl^−^ might be a risk factor for mortality among patients with severe positive FB (> 10%) is our most interesting finding. This result is consistent with the findings of a similar previous study that reported an association of an increase in the Cl^−^ load with mortality among patients receiving large-volume resuscitation [21]. These results suggest that the Cl^−^ load may have a significant impact in patients receiving large-volume fluid resuscitation. Second, we demonstrated no association of a positive fluctuation in Cl^−^ with 30-day mortality among patients with hypochloremia upon ICU admission. By contrast, a previous study by Oh and colleagues reported that an increase in the Cl^−^ level was associated with a decrease of mortality among hypochloremic patients with sepsis or septic shock [22]. Our study and the study by Oh and colleagues differed with respect to focus. Specifically, we focused on a mixed ICU population, whereas Oh and colleagues analyzed only patients with sepsis or septic shock who might have tended to present with more severe conditions. This difference in patient populations might have caused the discrepancy in the impact of positive fluctuations in Cl^−^ on mortality among critically ill patients. Future studies are needed to confirm the impact of the Cl^−^ load on mortality in patients with hypochloremia.

Our findings regarding the effects of a negative fluctuation in Cl^−^ are novel and interesting, as no previous study had evaluated the impact of a decreased Cl^−^ level after ICU admission on mortality. We found that a negative fluctuation in Cl^−^ might be a risk factor for 30-day mortality in patients with a negative FB (< 5%) or hypochloremia upon ICU admission. Consistent with previous studies, hypochloremia upon ICU admission was identified as an independent risk factor for mortality [23, 24]. Among patients in the cumulative negative FB group (< 0%), a negative fluctuation in Cl^−^ within 72 h after ICU admission suggested a significant body fluid loss without proper fluid replacement, which may have been consequent to septic shock, massive bleeding, or gastrointestinal losses. Likewise, patients with hypochloremia upon ICU admission and a negative fluctuation in Cl^−^ within the subsequent 72-h period might have experienced insufficient fluid resuscitation. However, the information regarding this issue remains lacking, and further study is needed to confirm the relationship between a negative fluctuation in Cl^−^ and the outcomes of critically ill patients.

Another interesting point of note in this study is the establishment of a cut-off point of 10 mmol L^− 1^ for fluctuations in the Cl^−^ level within 72 h after ICU admission. This value may be used as a reference in studies of 30-day mortality after ICU admission. Neyra and colleagues reported that a 5-mmol L^− 1^ increase in the Cl^−^ level at 72 h after ICU admission was associated with a 1.37-fold increase in hospital mortality [3], while Shaw and colleagues reported that a cut-off of 10 mmol L^− 1^ (identical to our study cut-off value) was associated with increased hospital mortality in patients with systemic inflammatory response syndrome [3, 5]. Our study differed from those previous studies because our analysis included all general critically ill patients (including post-surgical patients) and demonstrated associations between dyschloremia and 30-day mortality using both positive and negative fluctuations in Cl^−^ levels, respectively. From a statistical perspective, the optimal cut-off points for continuous variables could be determined using a cubic spline approach to maximize the impact on outcomes [25]. Therefore, we used 10 mmol L^− 1^ as a cut-off point because this was very near the endpoints of the U-shaped curves of 30-day mortality in our cubic spline analyses (Additional file 3: Figure S1 and Additional file 4: Figure S2).

This study had several limitations of note. First, it may have been subject to the selection bias characteristic of a retrospective cohort design. To minimize this bias, however, data collection and handling were performed by medical record technicians blinded to the purpose of the study. Second, the generalizability of our results may be limited by the single-center nature of the population and setting. Third, although we included the number of measurements within 72 h after ICU admission to reduce bias, our study was limited by the fact that the patients’ Cl^−^ levels were not measured at the same time point via the same method. Overall, a multicenter prospective study should be performed in future to confirm our findings.

## Conclusions

This study has demonstrated that a positive fluctuation in the Cl^−^ levels within the first 72 h of an ICU stay is associated with increased 30-day mortality among critically ill patients with a severe positive cumulative FB (> 10%), normochloremia, or hyperchloremia at the time of ICU admission. Additionally, a negative fluctuation in the Cl^−^ level during the first 72 h of an ICU stay was associated with increased 30-day mortality among critically ill patients with a negative cumulative FB (< 0%) or hypochloremia upon ICU admission.

## Additional files

Additional file 1: **Table S1**. Univariable Cox regression analysis of covariates for 30-day mortality after ICU admission. (DOCX 19 kb)

Additional file 2: **Table S2**. Interactions between fluctuations in Cl- levels with the associations of cumulative FB and dyschloremia upon ICU admission with 30-day mortality in a multivariable Cox regression analysis. (DOCX 18 kb)

Additional file 3: **Figure S1**. Restricted cubic spline analysis between 30-day mortality and a positive fluctuation in Cl^−^ within 72 h. (TIF 35 kb)

Additional file 4: **Figure S2**. Restricted cubic spline analysis between 30-day mortality and a negative fluctuation in Cl^−^ within 72 h. (TIF 38 kb)

## Abbreviations

Cl: Serum chloride
ICU: Intensive care unit
FB: Fluid balance
IRB: Institutional review board
HR: Hazard ratio
CI: Confidence interval
