# Supplementary material for: Association between fluctuations in serum chloride levels and 30-day mortality among critically ill patients: a retrospective analysis
Source: BMC Anesthesiol. 2019 May 17;19:79. doi: 10.1186/s12871-019-0753-3 (PMC6525376; doi:10.1186/s12871-019-0753-3)
Supplement: Supplementary file 1 — Table S1. Univariable Cox regression analysis of covariates for 30-day mortality after ICU admission. (DOCX 19 kb) [file 12871_2019_753_MOESM1_ESM.docx]

Table S1. Univariable cox regression analysis of covariates for 30-day mortality after ICU admission

| Variables | | | Univariable Cox model | |
| --- | --- | --- | --- | --- |
|  |  |  | Hazard ratio (95% CI) | *P*-value |
| Sex: male | | | 1.24 (1.08-1.42) | 0.003 |
| Age, year | | | 1.03 (1.02-1.03) | <0.001 |
| Body mass index, kg m^-2^ | | | 0.91 (0.89-0.93) | <0.001 |
| Comorbidities at ICU admission | | |  |  |
|  | APACHE II | | 1.08 (1.07-1.08) | <0.001 |
|  | Hypertension | | 0.94 (0.82-1.08) | 0.401 |
|  | Diabetes mellitus | | 1.48 (1.21-1.81) | <0.001 |
|  | Ischemic heart disease | | 0.94 (0.60-1.48) | 0.789 |
|  | Cerebrovascular disease | | 1.04 (0.76-1.43) | 0.822 |
|  | Chronic obstructive lung disease | | 1.18 (0.87-1.60) | 0.283 |
|  | Liver disease (LC, hepatitis, fatty liver) | | 2.37 (1.83-3.09) | <0.001 |
|  | Dyslipidaemia | | 0.52 (0.36-0.75) | <0.001 |
|  | Chronic kidney disease | | 2.56 (2.22-2.95) | <0.001 |
|  | Anaemia (Hb < 10 g dl^-1^) | | 3.77 (3.26-4.35) | <0.001 |
|  | Cancer | | 2.02 (1.75-2.32) | <0.001 |
| Characteristics of ICU admission | | |  |  |
|  | pRBC transfusion within 72h after ICU adm | | 2.08 (1.81-2.38) | <0.001 |
|  | Vasopressor infusion within 72h after ICU adm | | 2.03 (1.75-2.34) | <0.001 |
|  | RRT within 72h after ICU adm | | 2.16 (1.58-2.93) | <0.001 |
|  | ^a^fluid balance within 72 h after ICU adm | |  |  |
|  |  | 1% increase | 1.10 (1.09-1.10) | <0.001 |
|  |  | Even (0-5 %) | 1 | (<0.001) |
|  |  | Negative (< 0 %) | 1.26 (1.01-1.57) | 0.038 |
|  |  | Positive: Mild to moderate (5-10 %) | 2.58 (2.16-3.08) | <0.001 |
|  |  | Positive: Severe (>10 %) | 8.28 (6.93-9.90) | <0.001 |
|  | Fluid administration for 72h after ICU admission | |  |  |
|  |  | NaCl 0.9%, per 100 ml | 1.00 (1.00, 1.01) | <0.001 |
|  |  | Balanced crystalloid, per 100 ml | 0.97 (0.95, 0.97) | <0.001 |
|  |  | Hydroxyethyl starch, per 100 ml | 1.05 (1.03, 1.07) | <0.001 |
|  | The number of measurements for 72 h after ICU adm | | 1.46 (1.34, 1.59) | <0.001 |
|  | Admission through emergency department | | 2.85 (2.41, 3.36) | <0.001 |
|  | Admission department | |  |  |
|  | | Internal medicine or emergency medicine | 1 | (<0.001) |
|  | | Neurologic center | 0.15 (0.11-0.19) | <0.001 |
|  | | Post-cardiothoracic surgery | 0.35 (0.29-0.41) | <0.001 |
|  | | Post-other surgery | 0.38 (0.32-0.45) | <0.001 |

a: Cumulative fluid balance: (Total input fluid – Total output fluid ) in liter x 100 x weight in admission (kg)^-1^

ICU, intensive care unit; APACHE, acute physiology and chronic health evaluation; LC, liver cirrhosis; Hb, haemoglobin; pRBC, packed red blood cell; RRT, renal replacement therapy
