# Supplementary material for: Association between fluctuations in serum chloride levels and 30-day mortality among critically ill patients: a retrospective analysis
Source: BMC Anesthesiol. 2019 May 17;19:79. doi: 10.1186/s12871-019-0753-3 (PMC6525376; doi:10.1186/s12871-019-0753-3)
Supplement: Supplementary file 2 — Table S2. Interactions between fluctuations in Cl- levels with the associations of cumulative FB and dyschloremia upon ICU admission with 30-day mortality in a multivariable Cox regression analysis. (DOCX 18 kb) [file 12871_2019_753_MOESM2_ESM.docx]

Table S2. Interactions between fluctuation of Cl^-^ with cumulative FB groups and dyschloraemia on ICU admission for 30-day mortality in multivariable Cox regression analysis.

| Variables | | Multivariable model | |
| --- | --- | --- | --- |
|  |  | Hazard ratio (95% CI) | *P-value* |
| Interaction: ^a^Positive fluctuation of Cl^-^ *cumulative FB group^b^ | |  |  |
|  | ^a^Positive fluctuation of Cl^-^ ≤ 10 mmol L^-1^ * Even (0-5 %) | 1 | (0.149) |
|  | ^a^Positive fluctuation of Cl^-^ > 10 mmol L^-^ * Negative (< 0 %) | 0.75 (0.35, 1.61) | 0.454 |
|  | ^a^Positive fluctuation of Cl^-^ > 10 mmol L^-^ * Positive: Mild to moderate (5-10 %) | 0.51 (0.27, 0.95) | 0.034 |
|  | ^a^Positive fluctuation of Cl^-^ > 10 mmol L^-^ * Positive: Severe (>10 %) | 0.93 (0.53, 1.62) | 0.788 |
| Interaction: Negative fluctuation of Cl^-^ *cumulative FB group^b^ | |  |  |
|  | ^b^Negative fluctuation of Cl^-^ ≤ 10 mmol L^-1^ * Even (0-5 %) | 1 | (0.042) |
|  | ^b^Negative fluctuation of Cl^-^ > 10 mmol L^-^ * Negative (< 0 %) | 3.03 (1.00, 9.18) | 0.050 |
|  | ^b^Negative fluctuation of Cl^-^ > 10 mmol L^-^ * Positive: Mild to moderate (5-10 %) | 0.73 (0.25, 2.08) | 0.551 |
|  | ^b^Negative fluctuation of Cl^-^ > 10 mmol L^-^ * Positive: Severe (>10 %) | 0.96 (0.38, 2.39) | 0.925 |
| ^a^Positive fluctuation of Cl^-^ * Dyschloraemia at ICU adm | |  |  |
|  | ^a^Positive fluctuation of Cl^-^ ≤ 10 mmol L^-1^ * Normochloraemia at ICU adm | 1 | (0.005) |
|  | ^a^Positive fluctuation of Cl^-^ > 10 mmol L^-^ * Hypochloraemia at ICU adm | 0.42 (0.25, 0.73) | 0.002 |
|  | ^a^Positive fluctuation of Cl^-^ > 10 mmol L^-^ * Hyperchloraemia at ICU adm | 0.81 (0.45, 1.46) | 0.485 |
| ^b^Negative fluctuation of Cl^-^ * Dyschloraemia at ICU adm | |  |  |
|  | ^b^Negative fluctuation of Cl^-^ > 10 mmol L^-^ * Normochloraemia at ICU adm | 1 | (0.052) |
|  | ^b^Negative fluctuation of Cl^-^ > 10 mmol L^-^ * Hypochloraemia at ICU adm | 4.87 (1.31, 18.10) | 0.018 |
|  | ^b^Negative fluctuation of Cl^-^ > 10 mmol L^-^ * Hyperchloraemia at ICU adm | 1.50 (0.77, 2.91) | 0.229 |

a: Positive fluctuation of Cl^-^: (Maximum Cl^-^ – Preadmission Cl^-^) for 72 hours after ICU admission

b: Negative fluctuation of Cl^-^: (Preadmission Cl^-^ – Minimum Cl^-^) for 72 hours after ICU admission

c: Cumulative fluid balance (%): (Total input fluid – total output fluid, L) x 100 x weight at admission (kg)^-1^

FB, fluid balance; ICU, intensive care unit
