# Supplementary figures and images for: Association between fluctuations in serum chloride levels and 30-day mortality among critically ill patients: a retrospective analysis
Source: BMC Anesthesiol. 2019 May 17;19:79. doi: 10.1186/s12871-019-0753-3 (PMC6525376; doi:10.1186/s12871-019-0753-3)

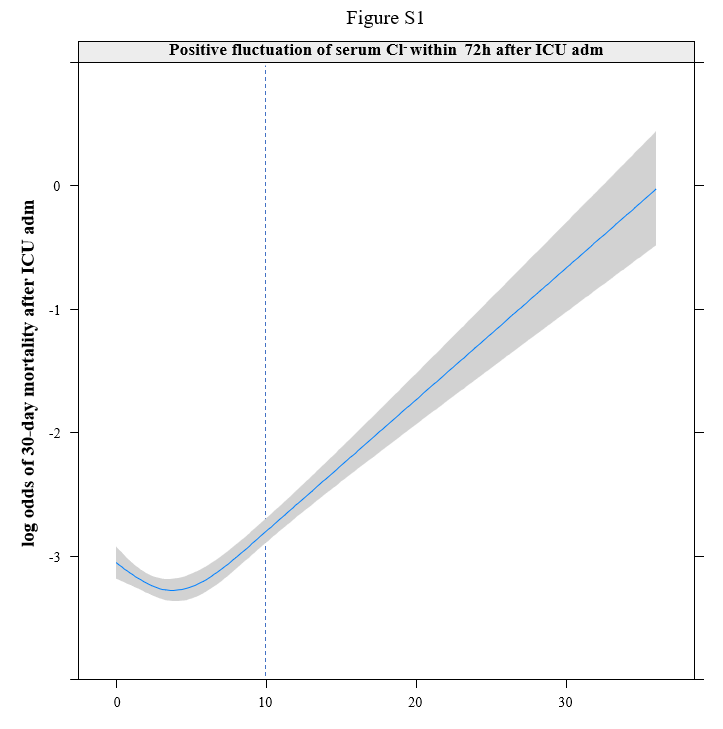

Supplement: Supplementary file 3 — Figure S1. Restricted cubic spline analysis between 30-day mortality and a positive fluctuation in Cl− within 72 h. (TIF 35 kb) [file 12871_2019_753_MOESM3_ESM.tif]

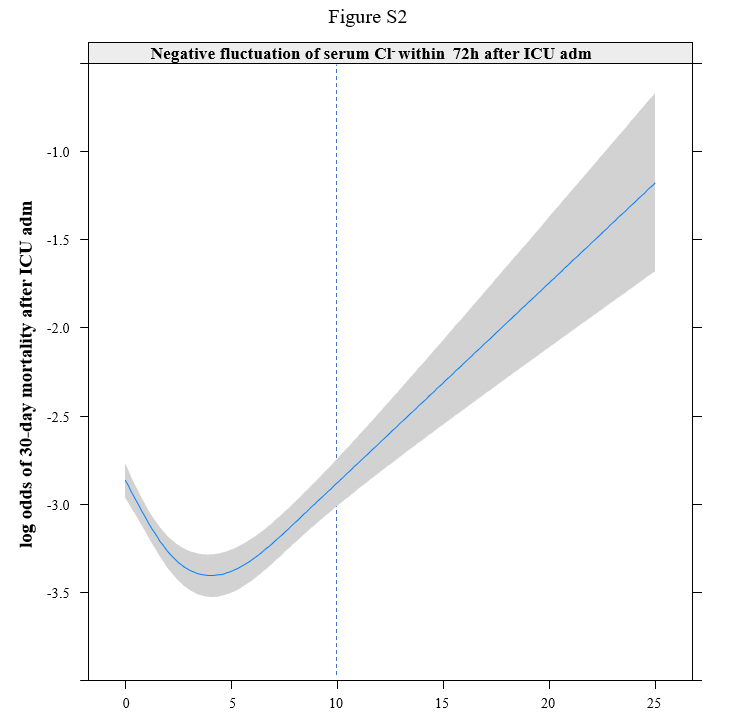

Supplement: Supplementary file 4 — Figure S2. Restricted cubic spline analysis between 30-day mortality and a negative fluctuation in Cl− within 72 h. (TIF 38 kb) [file 12871_2019_753_MOESM4_ESM.tif]
